# Investigation into the influence of mild hypothermia on regulating ferroptosis through the P53-SLC7A11/GPX4 signaling pathway in sepsis-induced acute lung injury

**DOI:** 10.1186/s40635-025-00713-3

**Published:** 2025-01-15

**Authors:** Liujun Tao, Jie Xu, Liangyan Jiang, Juntao Hu, Zhanhong Tang

**Affiliations:** Intensive Care Unit, The First Affiliated Hospital of Guangxi Medical University, No.6 Shuangyong Road, Nanning, 530021 Guangxi China

**Keywords:** Mild hypothermia, P53-SLC7A11/GPX4 signaling, Ferroptosis, Sepsis-induced acute lung injury

## Abstract

**Background:**

Sepsis-induced acute lung injury (S-ALI) significantly contributes to unfavorable clinical outcomes. Emerging evidence suggests a novel role for ferroptosis in the pathophysiology of ALI, though the precise mechanisms remain unclear. Mild hypothermia (32–34 °C) has been shown to inhibit inflammatory responses, reduce oxidative stress, and regulate metabolic processes. P53 has been reported to downregulate the transcriptional activity of solute carrier family 7 member 11 (SLC7A11), thereby limiting cystine uptake. This reduction in cystine availability compromises the activity of Glutathione peroxidase 4 (GPX4), a cystine-dependent enzyme, ultimately increasing cellular susceptibility to ferroptosis. However, it remains unclear whether mild hypothermia exerts protective effects through the P53-SLC7A11/GPX4 signaling pathway. This study investigates the influence of mild hypothermia on ferroptosis mediated by the P53-SLC7A11/GPX4 pathway in S-ALI.

**Methods:**

This study utilized both in vivo and in vitro models. In the vivo model, 64 Sprague–Dawley rats were employed, with 40 analyzed for survival outcomes. Sepsis was induced using the cecum ligation and puncture (CLP) method, after which rats were subjected to either normothermic (36–38 °C) or mild hypothermic (32–34 °C) conditions for a duration of 10 h. Twelve hours post-surgery, blood samples, bronchoalveolar lavage fluid, and lung tissue samples were harvested for histological analysis, measurement of inflammatory markers, wet/dry ratios, blood gas analysis, assessment of oxidative stress and ferroptosis, Western blotting, and RT-qPCR analysis. In the in vitro model, RLE-6TN cells were exposed to lipopolysaccharide (LPS) for 24 h under normothermic and mild hypothermic conditions. These cells were then evaluated for cell viability, inflammatory markers, oxidative stress levels, ferroptosis markers, as well as Western blot and RT-qPCR analyses.

**Results:**

CLP-induced sepsis led to elevated levels of inflammatory markers, increased lung injury scores, and heightened oxidative stress markers. These detrimental effects were significantly ameliorated by mild hypothermia. Furthermore, mild hypothermia reversed the modified expression of P53, SLC7A11, and GPX4 signaling molecules. Notably, mild hypothermia also improved the 5-day survival rate of CLP rats.

**Conclusion:**

Mild hypothermia attenuates S-ALI and modulates ferroptosis through the P53-SLC7A11/GPX4 signaling pathway.

**Supplementary Information:**

The online version contains supplementary material available at 10.1186/s40635-025-00713-3.

## Background

Acute lung injury (ALI) is a syndrome resulting from both intrinsic lung factors and external influences [1–3]. Sepsis is a critical medical condition marked by profound organ dysfunction and an impaired host response to infection, posing a significant threat to patient survival in clinical settings [4]. During sepsis, the lungs are highly vulnerable, being the most frequently failing organ [5]. Sepsis-induced acute lung injury (S-ALI) arises from an infection-induced immune response that leads to lung tissue damage and dysfunction, thereby increasing the risk of mortality [6]. The pathogenesis of ALI is complex, involving cytotoxic mechanisms such as inflammation and oxidative stress. Despite this understanding, effective targeted treatments remain insufficiently developed [7].

Mild hypothermia (32–34 °C) has been demonstrated to inhibit inflammatory responses, reduce oxidative stress, and regulate metabolic processes, thereby mitigating multiorgan damage to the heart [8], liver [9], lungs [10], kidneys [11], and brain [12]. Additionally, according to certain research, people with sepsis who experience mild hypothermia have a better early prognosis [13, 14]. Similarly, the overall survival rate in animal models of sepsis increases following mild hypothermia treatment [15, 16]. However, hypothermia has been reported to significantly increase the risk of cardiac arrhythmias and thrombocytopenia [17, 18]. Additionally, a substantial randomized controlled study found that Induced hypothermia has shown no significant reduction in mortality among patients with septic shock and is therefore not recommended for use in this patient population [19]. Further research is required to elucidate the potential role of hypothermia in this context and to gain a deeper understanding of the underlying mechanisms.

In contrast to apoptosis, ferroptosis is a unique form of iron-dependent programmed cell death, aggravates oxidative stress reactions [20]. Research has indicated that genes associated with ferroptosis are involved in lipopolysaccharide (LPS)-induced ALI [21],and multiple investigations have established a strong association between ferroptosis and ALI [22]. Ferroptosis plays a pivotal role in S-ALI [23]. Evidence from S-ALI models highlights the involvement of lung cell ferroptosis, suggesting that targeting ferroptosis could be a therapeutic strategy for ALI [24, 25]. Solute carrier family 7 member 11 (SLC7A11) transports the precursor of glutathione (a key molecule in antioxidant stress), cysteine Glutathione (GSH), into the cytoplasm and is a vital gene participates in ferroptosis [26, 27]. Similarly, Glutathione peroxidase 4 (GPX4) is an irreplaceable enzyme in the GPX family that directly reduces and neutralizes lipid hydroperoxides, making it another essential gene in ferroptosis. A significant part of the ferroptosis antioxidant system is played by GSH and GPX4 [28]. GSH serves as the primary cellular antioxidant and as a cofactor for GPX4. GSH, through the catalysis of GPX4, GSH effectively reduces the buildup of reactive oxygen species (ROS) in cells, thereby preventing the initiation of ferroptosis [29]. Additionally, P53 can suppress cystine uptake through the Xc⁻ system by reducing the expression of SLC7A11, which in turn impacts GPX4 activity. This reduces the body's antioxidant capacity, leading to elevated ROS levels and ultimately inducing ferroptosis[30, 31]. Consequently, the P53-SLC7A11/GPX4 pathway may represent a promising therapeutic target for counteracting ferroptosis in ALI.

However, the potential of hypothermia to protect lung tissue from ferroptosis by modulating the P53-SLC7A11/GPX4 signaling pathway remains uncertain. To address this gap, the present study investigates the safeguarding effect of hypothermia in S-ALI and its interaction with the P53-SLC7A11/GPX4 signaling pathway.

## Materials and methods

**Fig. 1:**
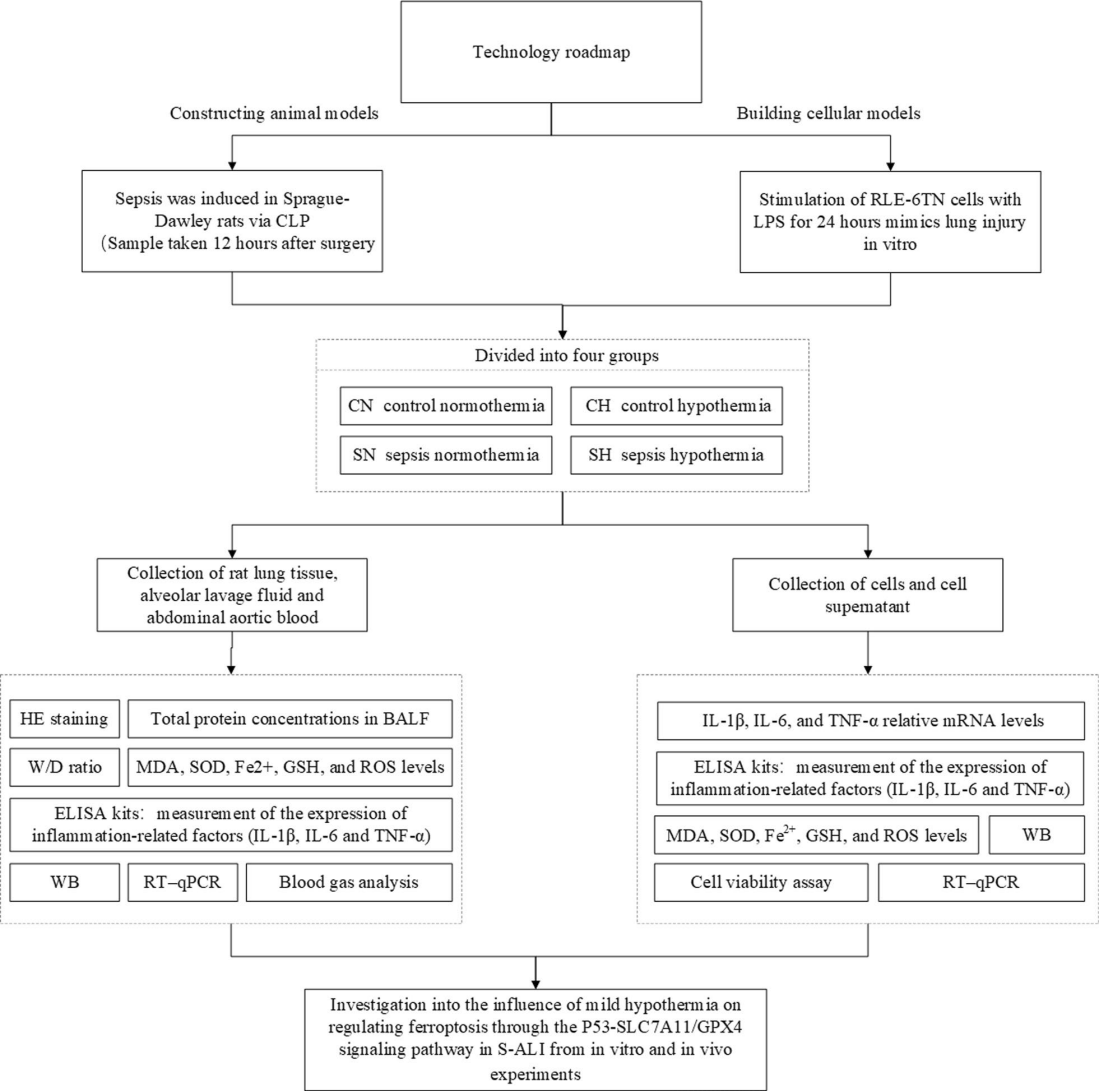
Technical roadmap for in vivo and in vitro experiments

The whole relative procedures were performed as per the flowchart (Fig. 1).

### Ethical approval and experimental animals

This study received ethical approval from the Animal Ethics Committee of Guangxi Medical University (Approval No. 202306672). Animal handling adhered to the guidelines of the Chinese Experimental Animal System Ethics Review Committee. The required sample size was calculated using a resource equation method [32, 33]. We utilized 64 male, 8-week-old Sprague–Dawley rats (220–300 g) sourced from Guangxi Medical University Experimental Animal Center. The rats were randomly distributed into subgroups from the numbered cohort.

### Rat model of cecal ligation and puncture (CLP)

Rats were fasted for 12 h with access to water prior to modeling. Sepsis was induced using the CLP method [34, 35]. Prior to surgery, rats were weighed and anesthetized with 3% sodium pentobarbital (50 mg/kg). The depth of anesthesia was monitored by applying pressure to the toe with forceps; adequate anesthesia was confirmed by the absence of limb withdrawal (e.g., no limb flexion). The abdomen was prepared by shaving and iodine disinfection. A 3 cm midline incision was made to access the cecum, which was ligated near the ileocecal valve and punctured with an 18G needle to release minimal intestinal content. The cecum was returned to the abdominal cavity, and the abdominal incision was closed in layers. Sham operations involved abdominal surgery without cecum ligation or puncture. Postoperatively, subcutaneous saline was administered to prevent shock.

### Targeted temperature control

For rats in the hypothermic group, ice packs and an ALC-HTP animal temperature control system (Shanghai Alcott Biotech Co. Ltd., China) were used to rapidly lower body temperature and maintain rectal temperature between 32 °C and 34 °C. The ALC-HTP system’s animal-specific thermometer, which has a digital display, was utilized to measure rectal temperature. The target temperature was achieved within approximately 10 min. After 10 h of intervention, thermal blankets were used to gradually restore body temperature at a rate of 0.2–0.5 °C per hour, aiming for a temperature of 36–38 °C. The rectal temperature of the normothermic group was maintained between 36 °C and 38 °C. Anesthesia was sustained during temperature control through hourly intraperitoneal injections of 3% sodium pentobarbital (15 mg/kg).

### Experimental design and grouping

Sixty-four male SD rats were used in total. To ensure that our study groups were allocated in a manner that was both fair and unbiased, we utilized a computer-generated random number table. Each animal was assigned a unique number, and a random number generator was used to determine the group allocation. Of these, 24 rats were randomly allocated into four groups: (1) control normothermia (CN), (2) control hypothermia (CH), (3) sepsis normothermia (SN), and (4) sepsis hypothermia (SH), with six rats per group. Twelve hours post-CLP, the rats were euthanized, and samples were collected for further analysis.

The remaining 40 rats were assigned to four groups of ten each for survival monitoring over a 5-day period. With hourly observations on the first day post-CLP, two-hourly assessments on the second and third days, and every 6 h on the following 2 days. The survival data were analyzed following the 5-day observation period.

### Evaluation of sepsis models

Referring to the evaluation indexes of sepsis animal model published by Bradly Shruma et al [36], the success of sepsis modeling was comprehensively assessed using seven indices: the animal's appearance, level of consciousness, activity, response to stimulus, eye opening, respiration rate, and respiratory quality, In our experiments, CLP-operated septic rats exhibited erect hair, arched backs, reduced activity, depressed spirits, weakened or absent responses to stimuli, closed eyes with insensitivity to pain stimulation and crust-like corners, and increased respiratory rates. Upon opening the abdominal cavity, obvious intestinal dilatation, edema, congestion; ligated cecum with gray coloration or necrosis; mesenteric adhesion and pus moss formation; and the presence of large amounts of bloody, turbid ascites accompanied by a foul odor were observed. The lungs of sham-operated rats were elastic, smooth, and light red, whereas the lungs of septic rats were dark in color with obvious congestion, edema, and local hemorrhage, indicating successful induction of sepsis manifestations in rats.

### Hematoxylin and eosin (H&E) staining

Lung tissues were fixed in 4% paraformaldehyde for 24 h, dehydrated with ethanol, and embedded in paraffin. Thin sections were prepared and stained with HE for histological examination under a light microscope. Lung injury was assessed using scoring criteria from the American Thoracic Society's official report [35]. Detailed methodologies can be found in the supplementary materials.

### Bronchoalveolar lavage fluid (BALF) collection and analysis

The rats were anesthetized, and the trachea was exposed via a cervical incision to occlude the right bronchus. A small cut was then made in the trachea to insert the lavage tube into the left bronchus. Subsequently, 1 ml of ice-cold saline was infused through the catheter into the left lung, and BALF was collected by suctioning three times. Samples were centrifuged at 3000 rpm for 10 min at 4 ℃ and the supernatant was collected and stored at -80℃ protein concentration was measured using a BCA assay kit (Beyotime, Shanghai, China).

### Measurement of the wet/dry weight (W/D) ratio

To determine the severity of pulmonary edema, the W/D ratio was calculated. After successful modeling, the right upper lung was clipped and cleaned with PBS, and absorbent paper was used to remove surface moisture. The tissue was subsequently weighed to obtain wet weight (wet mass). After 48 h of oven drying, the tissue was weighed again to obtain the dry weight (dry mass). The W/D ratio was then computed.

### Blood gas analysis

Rats were anesthetized, and their limbs were properly fixed. The midline was utilized to cut the abdominal cavity, and the intestines were moved aside using cotton. The abdominal aorta was fully exposed, blood samples were collected and analyzed using a Blood Gas Analyzer Model ABL 90 (Radiometer Medical Devices Ltd, Denmark).

### Enzyme-linked immunosorbent assay (ELISA)

Interleukin-1β (IL-1β), interleukin 6 (IL-6), and tumor necrosis factor-α (TNF-α) levels were quantified using ELISA kits (FANKEW, Shanghai, China) according to the manufacturer's instructions. Absorbance at 450 nm was measured with a microplate reader (Huaan Magnech, Beijing, China).

### MDA, SOD, Fe^2+^, ROS, and GSH measurements

Malondialdehyde (MDA), superoxide dismutase (SOD), ferrous iron (Fe^2^⁺), ROS, and GSH levels were quantified using specific assay kits: MDA (A003-1-2, Nanjing Jiancheng Bioengineering Institute, China), SOD (BC5165, Solarbio, China), iron (BC5415, Solarbio, China), ROS (BB-475015, BestBio, China), and GSH (A006-2-1, Nanjing Jiancheng Bioengineering Institute, China). Assays were conducted following the manufacturer's protocols.

### Real-time quantitative PCR (RT-qPCR)

Total RNA was isolated using the RNAeasy™ Animal RNA Isolation Kit with Rotary Column (Beyotime, R0026), following the manufacturer's guidelines. RNA purity and concentration were assessed using a Nanodrop 2000 Ultra-Micro Spectrophotometer. Reverse transcription was carried out using the Takara RT kit (RR036A) according to the provided protocol, and the cDNA products were stored at − 20 °C. The TB Green PCR Kit (Takara, RR820A) was used for RT-qPCR, and relative mRNA expression was quantified using β-actin as an internal control.

### Western blot analyses

Western blotting was utilized to evaluate the expression of P53, SLC7A11, and GPX4 proteins. Samples were stored at – 80 °C prior to protein extraction, which was carried out using RIPA lysis buffer (Beyotime, Shanghai, China). Extracted proteins were separated via SDS-PAGE and transferred onto a PVDF membrane (Millipore, USA). After blocking with 5% skim milk, the membrane was rinsed with TBST and incubated overnight at 4 °C with primary antibodies targeting P53 (1:5000, Proteintech), SLC7A11 (1:500, Zheneng Bio), GPX4 (1:500, Zheneng Bio), and β-actin (1:10,000, Zheneng Bio). The next day, after the membrane was removed from the primary antibody mixture, TBST was used to thoroughly rinse the membrane. The secondary antibody (1:10,000 in TBST, CST) was incubated for 1 h at room temperature, protected from light. Protein signals were visualized using the Li-Cor Odyssey Infrared Imaging System (Li-Cor, USA).

### Cell culture

RLE-6TN cells were cultured in RPMI 1640 medium supplemented with 10% fetal bovine serum (FBS, Oricell, USA) and antibiotics. Cells were maintained at 37 °C in a 5% CO_2_ incubator. In vitro sepsis was induced by exposing cells to LPS for 24 h.

### Cell viability assay

Cell viability was evaluated using the Cell Counting Kit-8(CCK-8) (Meilunbio, Dalian, China).Each well of a 96-well plate was seeded with 5,000 RLE-6TN cells and subsequently treated with LPS for 24 h. Groups were organized based on the experimental design: control normothermia (CN), control hypothermia (CH), sepsis normothermia (SN), and sepsis hypothermia (SH). The SN and SH groups were treated with varying concentrations of LPS for 24 h. Subsequently, the CH and SH groups were transferred to a separate incubator maintained at 33 °C. Cell viability was evaluated using a CCK-8 assay, where cells were incubated with a 1:10 dilution of CCK-8 solution for 1 h at 37 °C, and absorbance was recorded at 450 nm.

### ROS measurement in cells

RLE-6TN cells were incubated overnight with 10 µg/mL LPS. Subsequently, the cells were treated with 500 µL of a 10 µM DCFH-DA fluorescent probe and incubated for 30 min in the absence of light. To remove any remaining unattached probe, the cells were rinsed with PBS. Fluorescence intensity was measured using ImageJ software (XLIH, USA) following imaging with a Nikon Eclipse Ci-L microscope (Nikon, Japan).

### Statistical methods

Quantitative data are expressed as mean ± standard deviation. Data following a normal distribution were analyzed using one-way ANOVA, preceded by a homogeneity of variances test. For homogenous variances, pairwise comparisons were conducted using the least significant difference (LSD) test, while post hoc multiple comparisons utilized the Bonferroni correction. If variances were unequal, Welch's test was applied, followed by Dunnett's T3 test for multiple comparisons. For non-normally distributed data, the rank-sum test was performed. Survival time and survival rate of rats were assessed using Kaplan–Meier (K-M) analysis, and the log-rank test was employed for group comparisons. All statistical analyses were performed using the SPSS version 23.0 software (IBM Corp., Armonk, NY, USA). A *P*-value < 0.05 was considered statistically significant.

## Results

### Mild hypothermia attenuates CLP-induced inflammatory cytokine levels in rats

**Fig. 2:**
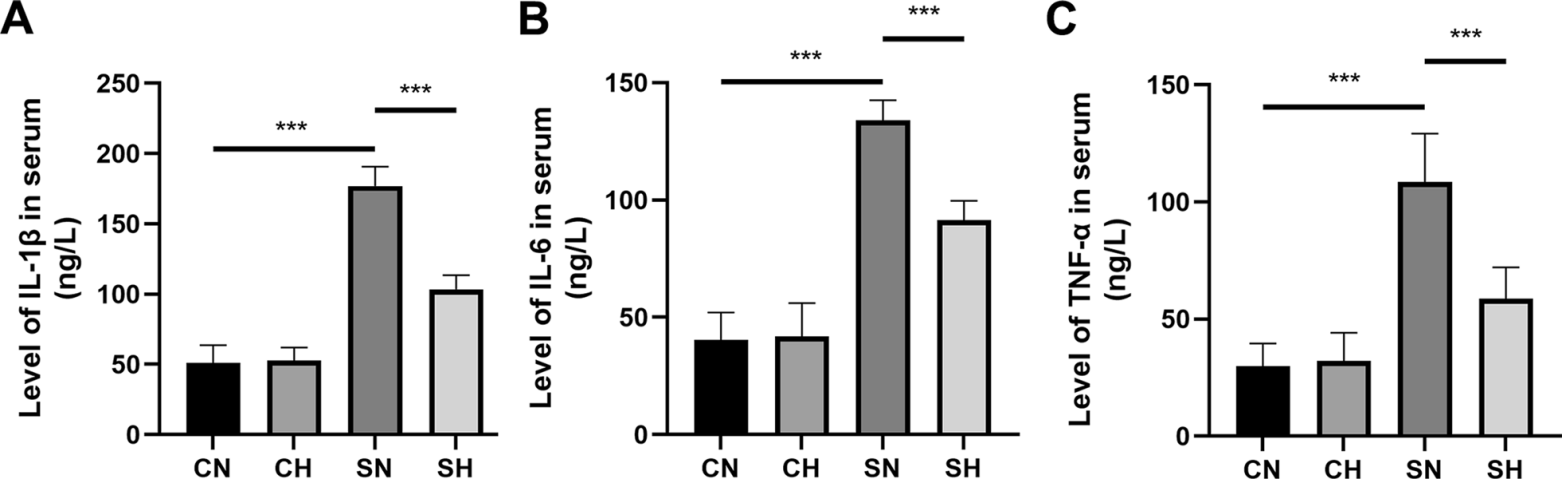
Mild hypothermia therapy reduced serum inflammatory factors in rats.** A**–**C** Display the ELISA results for serum levels of IL-1β, IL-6, and TNF-α (mean ± SD, *n* = 6) across the CN, CH, SH, and SN groups. *P < 0.05, **P < 0.01, and ***P < 0.001

Cytokine levels showed no significant differences between the CN and CH groups. In contrast, the SN group displayed a notable increase in cytokine levels relative to the CN group. Moreover, serum from SH rats exhibited significantly lower concentrations of IL-1β, IL-6, and TNF-α compared to the SN group (Fig. 2A–C).

### Mild hypothermia alleviates CLP-induced acute lung injury and improves survival rates in sepsis rats

**Fig. 3:**
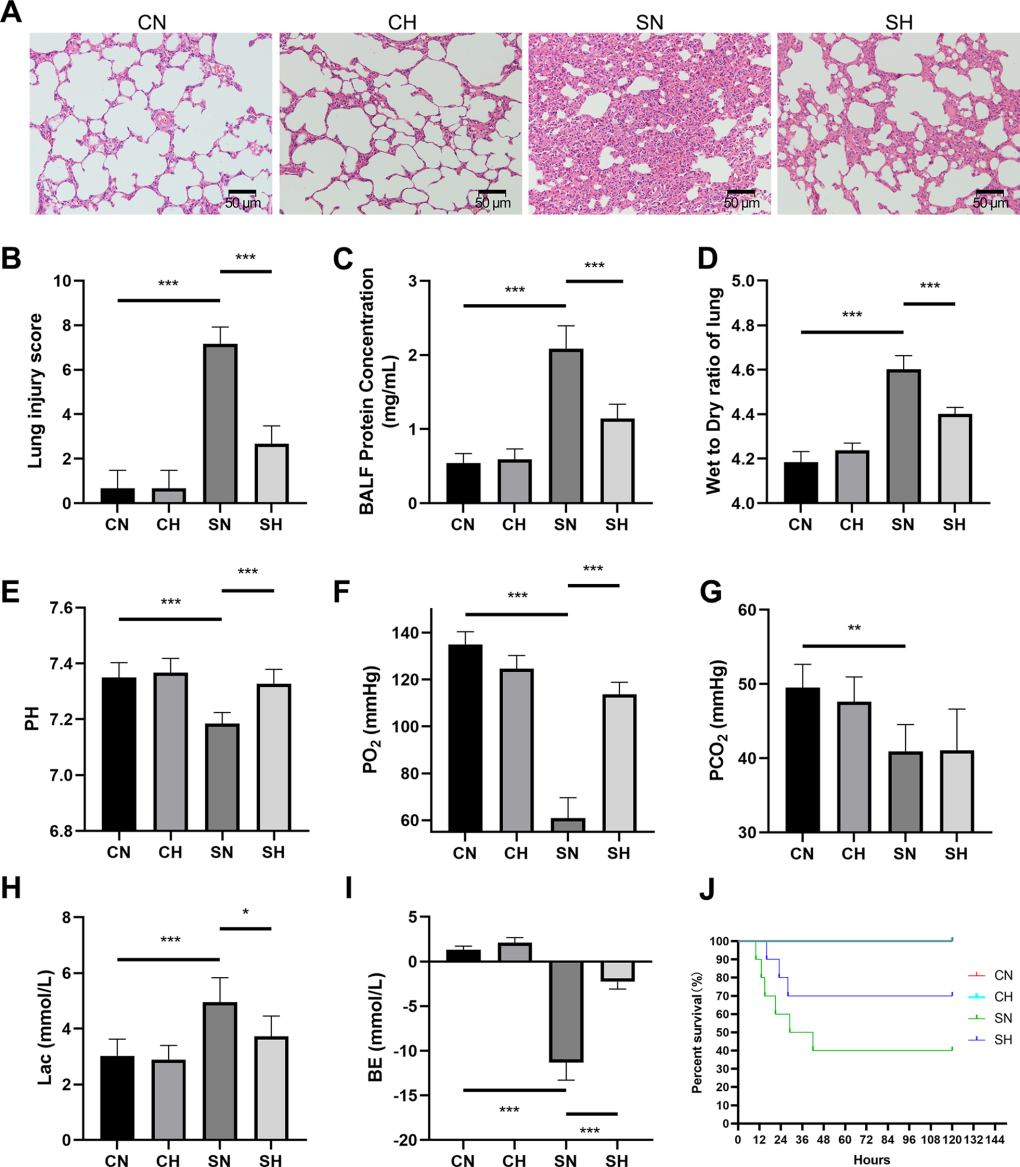
HE staining of lung tissue, total protein content in BALF and the W/D ratio, blood gas analysis, and survival analysis. **A** shows HE-stained lung tissue sections from CN, CH, SH, and SN groups (scale bar = 50 µm). **B** Presents the injury scores for the CN, CH, SH, and SN groups. **C** Displays the total protein content in each group's BALF, and **D** shows the W/D ratio for the lungs. **E**–**I** illustrate the pH (**E**), PO₂ (**F**), PCO₂ (**G**), Lac (**H**), and BE (**I**) values in bar charts (mean ± SD, *n* = 6). **J** Depicts the survival analysis results (mean ± SD, *n* = 10). *P < 0.05, **P < 0.01, and ***P < 0.001

First, histological changes were analyzed using light microscopy (Fig. 3A). The SN group displayed marked thickening of the alveolar septa and interstitial spaces, along with infiltration of inflammatory cells in both the airway and alveolar regions, and damage to the endothelial and epithelial layers when compared to the CN group. In contrast, these histological alterations were ameliorated in the SH group, as evidenced by the lung injury scores (Fig. 3B).

Second, lung barrier integrity was evaluated using the lung wet/dry weight ratio and BALF protein concentration. Both indicators increased significantly after CLP, whereas mild hypothermia effectively reduced these levels (Fig. 3C, D).

Additionally, arterial blood gas analysis revealed no significant differences in pH, PO_2_, PCO_2_, Lac, or BE between the CH and CN groups. Compared to the CN group, the SN group exhibited decreased pH, PO₂, PCO_2_, and BE, along with elevated Lac levels. In contrast, the SH group demonstrated improved pH, PO_2_, and BE, while Lac levels were reduced (Fig. 3E–I).

The impact of mild hypothermia on survival outcomes was also assessed. The results showed that there were no fatalities in either the CN or CH groups during the 5-day observation period. In contrast, the SN group exhibited a decreasing survival rate, while survival in the SH group was significantly extended (Fig. 3J).

### Mild hypothermia alleviates CLP-induced oxidative stress and ferroptosis in septic rats

**Fig. 4:**
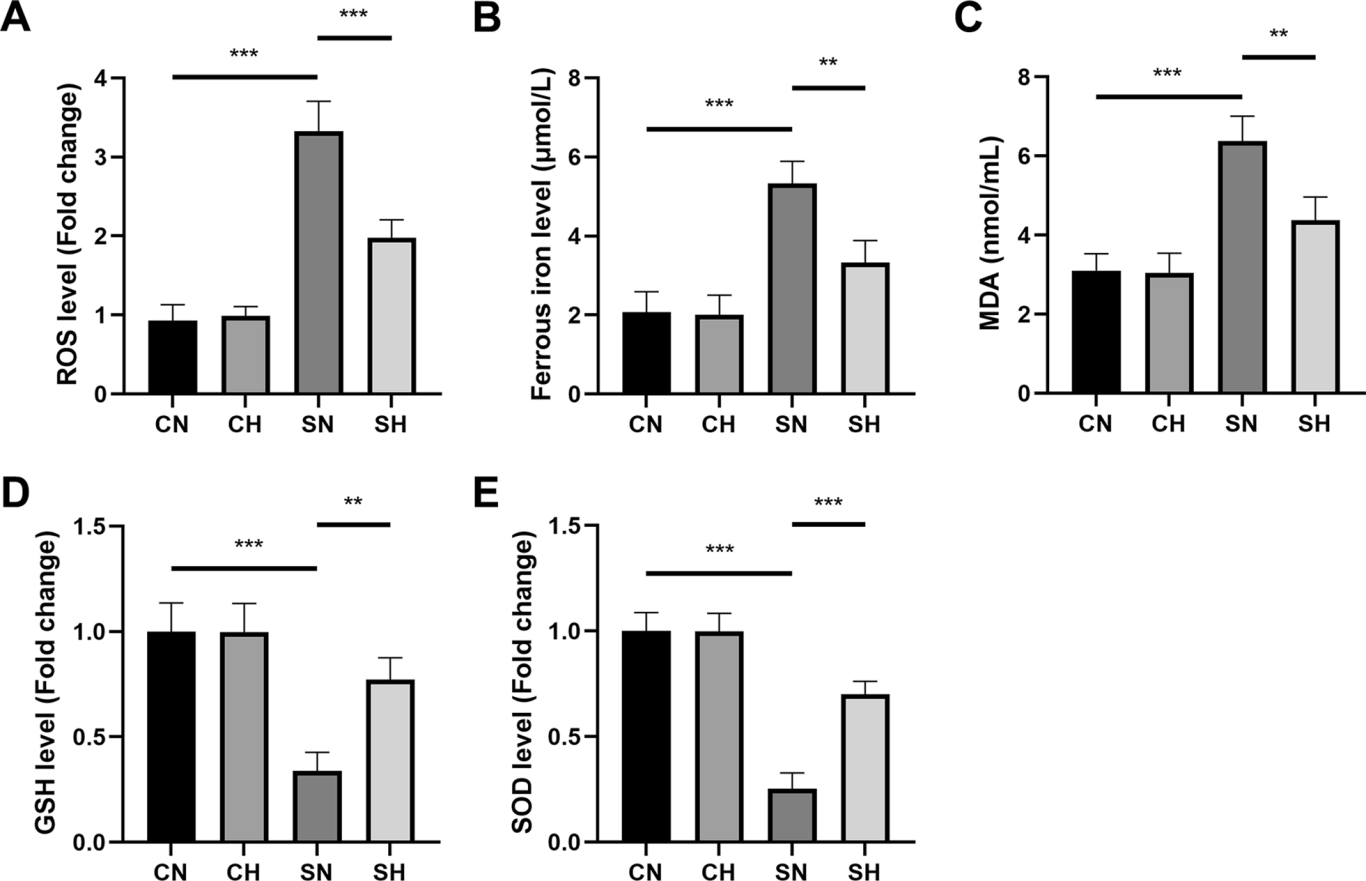
Levels of ROS, Fe^2^⁺, MDA, GSH, and SOD in rats. **A** Displays ROS levels. **B** Displays Fe^2^⁺ levels. **C** Displays MDA levels. **D** Displays GSH levels. **E** Displays SOD levels (mean ± SD, *n* = 3). *P < 0.05, **P < 0.01, and ***P < 0.001

The SN group exhibited elevated levels of ferroptosis markers (ROS, MDA, and Fe^2+^) and decreased antioxidant markers (SOD and GSH) in lung tissues compared to the CN group. Compared to the SN group, the SH group exhibited elevated SOD and GSH levels, along with decreased MDA, Fe^2+^, and ROS levels (Fig. 4A–E). These results suggest that ferroptosis and oxidative stress occur in the lung tissues of rats with CLP-induced sepsis. Moreover, mild hypothermia appears to enhance antioxidant capacity and mitigate ferroptosis in this model.

### Mild hypothermia regulates the P53-SLC7A11/GPX4 pathway to mitigate ferroptosis in septic rats

**Fig. 5:**
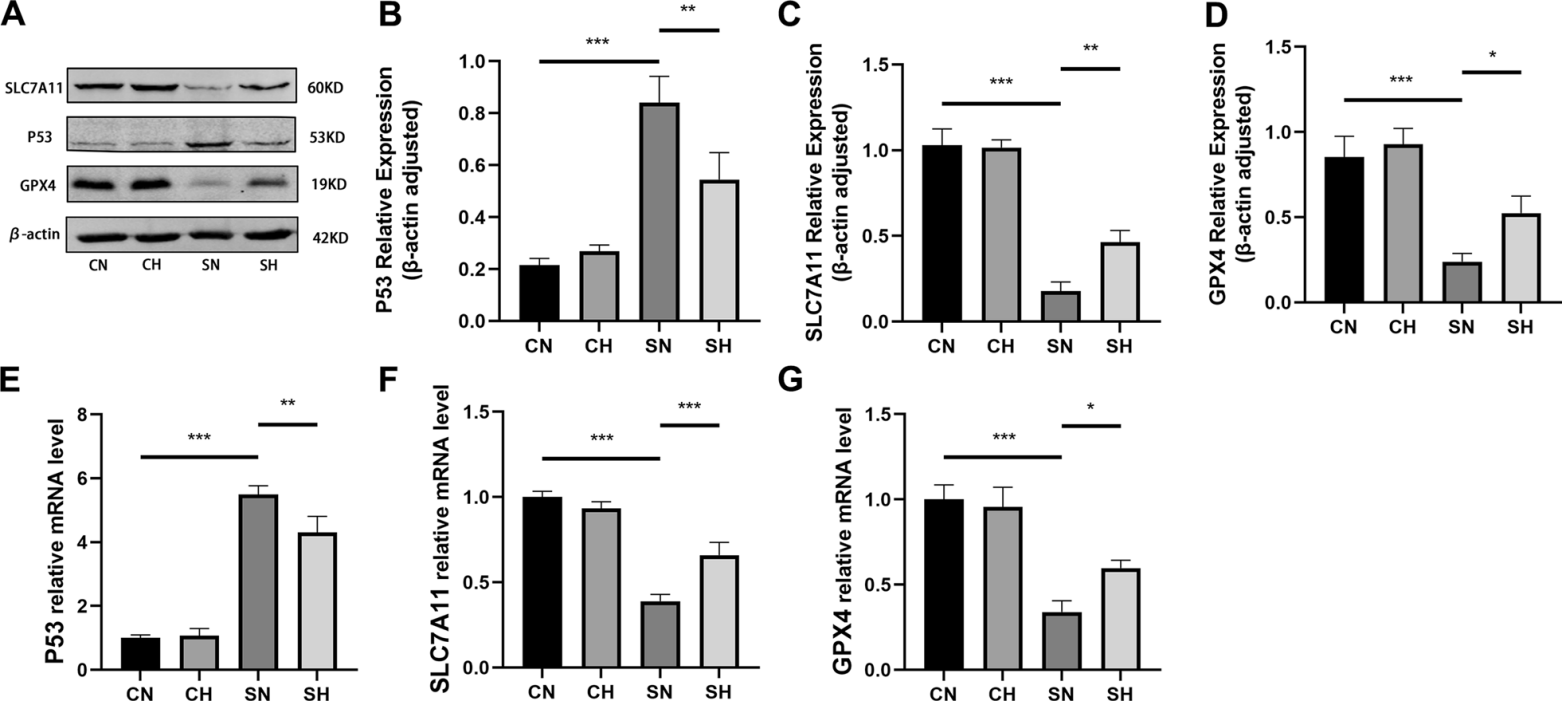
Protein and mRNA expression 12 h following CLP surgery in lung samples of rats. **A**–**D** show the expression levels of P53, SLC7A11, GPX4, and β-actin proteins, respectively. **E**–**G** Display the mRNA expression levels of P53, SLC7A11, GPX4, and β-actin, respectively (mean ± SD, *n* = 3). *P < 0.05, **P < 0.01, and ***P < 0.001

P53/SLC7A11/GPX4 has been proven to be a classic signaling pathway of ferroptosis [37, 38]. To evaluate the impact of mild hypothermia on this pathway, Western blot and RT-qPCR analyses were conducted. Compared to the CN group, the SN group exhibited decreased SLC7A11 and GPX4 expression at both the mRNA and protein levels, along with an upregulation of P53 expression. Conversely, the SH group showed reduced P53 expression at both the mRNA and protein levels, while SLC7A11 and GPX4 expression were significantly increased compared to the SN group (Fig. 5A–G).

### Mild hypothermia attenuates LPS-induced inflammatory response in RLE-6TN cells

**Fig. 6:**
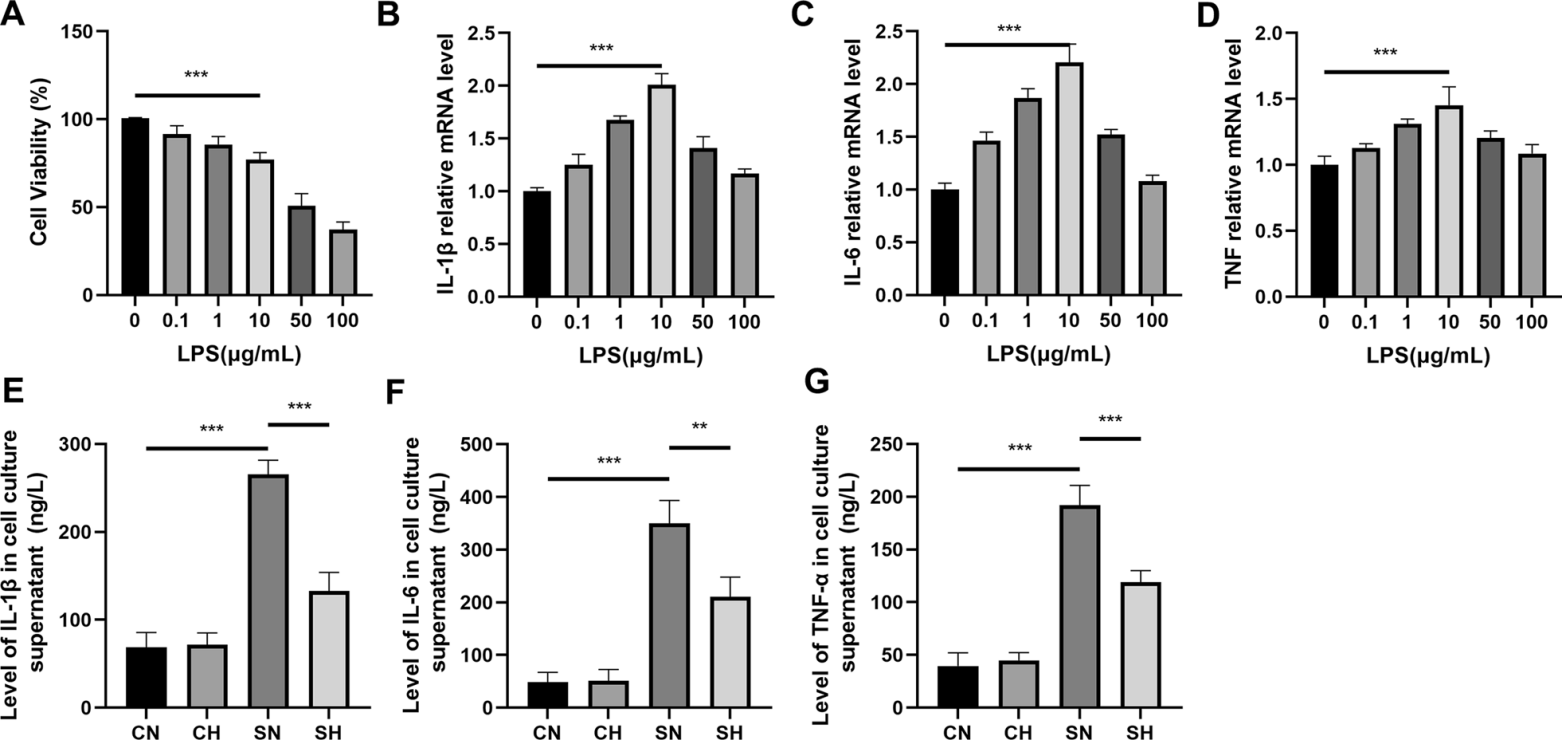
ELISA assays, inflammatory factor mRNA analysis for different LPS concentrations, and CCK-8 assays. **A** Shows the viability of RLE-6TN cells as determined by CCK-8 assays. **B**–**D** Display the mRNA levels of IL-1β, IL-6, and TNF-α, respectively. **E**–**G** Present the ELISA results for IL-1β, IL-6, and TNF-α levels (mean ± SD, *n* = 3). *P < 0.05, **P < 0.01, and ***P < 0.001

Initially, we determined the optimal concentration of LPS required to stimulate RLE-6TN cells in vitro. Cell viability were largely unaffected at LPS concentrations of 0, 0.1, 1, and 10 µg/mL, indicating minimal cytotoxicity within this range. However, a significant reduction in viability was observed at 50 µg/mL (Fig. 6A). qRT-PCR analysis revealed that mRNA expression levels of IL-1β, IL-6, and TNF-α reached their peak at an LPS concentration of 10 µg/mL (Fig. 6B–D). Based on these results, 10 µg/mL LPS was chosen to stimulate cells and establish the in vitro sepsis model.

ELISA analysis indicated that the SN group exhibited elevated levels of inflammatory cytokines, including IL-1β, IL-6, and TNF-α, compared to the CN group. In contrast, the SH group showed a significant reduction in these cytokine levels relative to the SN group (Fig. 6E–G).

### Mild hypothermia attenuates oxidative stress and ferroptosis in LPS-induced RLE-6TN cells

**Fig. 7:**
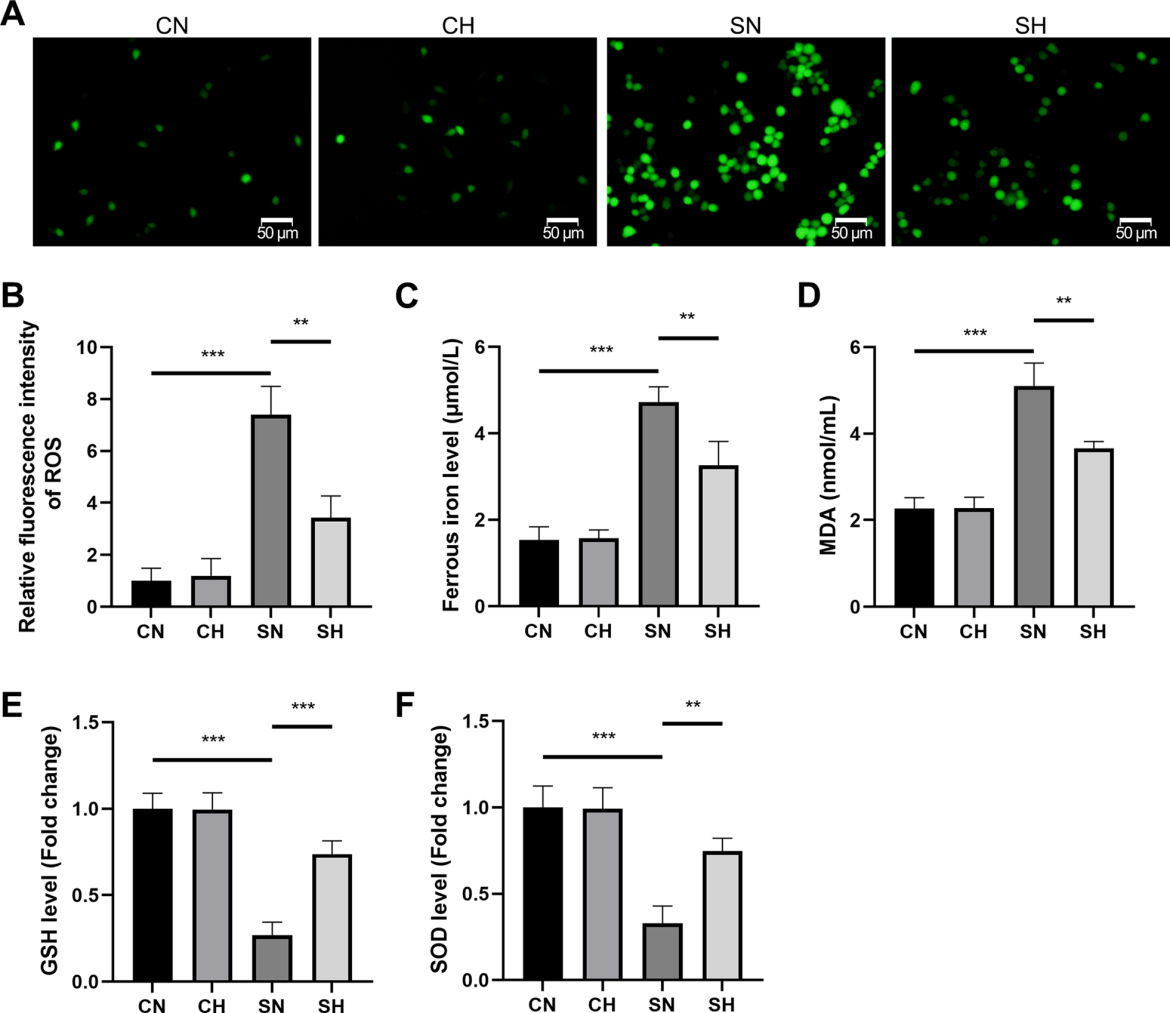
ROS, Fe^2+^, MDA, GSH, and SOD levels in cells. Under a microscope, **A** displays the amount of cellular ROS (scale bar = 50 µm). The statistical analysis of ROS fluorescence intensity is shown in **B**. **C** Shows Fe.^2^⁺ levels. **D** Shows MDA levels. **E** Shows GSH levels. **F** Presents SOD levels (mean ± SD, *n* = 3). *P < 0.05, **P < 0.01, and ***P < 0.001

Compared to the CN group, the SN group displayed reduced SOD and GSH levels, along with increased MDA, Fe^2+^, and ROS levels. In contrast, the SH group showed elevated SOD and GSH levels, while MDA, Fe^2+^, and ROS levels were significantly decreased compared to the SN group (Fig. 7A–F). These results suggest that oxidative stress and ferroptosis are induced in LPS-stimulated RLE-6TN cells, but mild hypothermia mitigates these effects.

### Mild hypothermia regulates the P53-SLC7A11/GPX4 pathway to mitigate ferroptosis in LPS-induced RLE-6TN cells

**Fig. 8:**
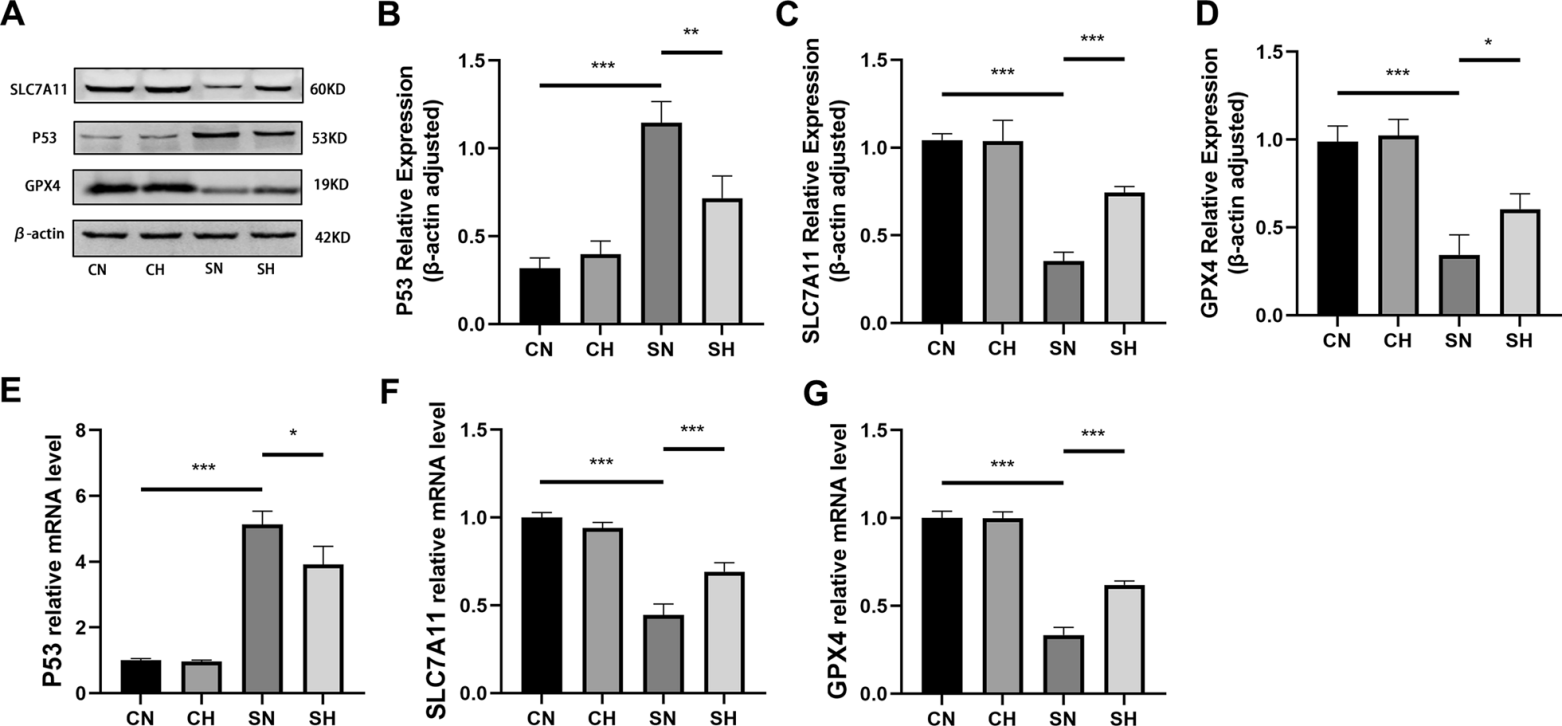
Protein and mRNA levels in cell samples obtained from the CN, CH, SH, and SN groups 24 h after LPS activation. **A**–**D** Illustrate the protein expression of P53, SLC7A11, GPX4, and β-actin, respectively, while panels **E**–**G** present the mRNA expression levels of P53, SLC7A11, and GPX4 (mean ± SD, *n* = 3). *P < 0.05, **P < 0.01, and ***P < 0.001

SLC7A11 and GPX4 levels were lower in the SN group in comparison to the CN group. However, the P53 protein and mRNA expression levels were relatively high. When contrasting the SN group, the SH group presented lower P53 protein and mRNA expression levels but greater SLC7A11 and GPX4 levels (Fig. 8A–G).

## Discussion

Severe trauma, infection, or surgery can all precipitate sepsis; as sepsis progresses, it may lead to shock and multiple organ dysfunction syndrome (MODS), with the lungs being particularly affected. The progression of sepsis involves increased ferroptosis in alveolar cells and heightened oxidative stress, both of which are pivotal in the development of pulmonary edema and lung tissue damage [39, 40]. Iron buildup and lipid peroxidation are key drivers of ferroptosis, a recently recognized form of cell death [41, 42]. Ferroptosis plays a significant role in organ damage associated with sepsis, including damage to the heart, liver, and kidneys. Numerous studies have demonstrated that ferroptosis occurs during sepsis, and inhibiting ferroptosis can mitigate the detrimental effects of sepsis on various organs [43, 44], suggesting that ferroptosis inhibition could be a promising strategy for organ protection. Strong evidence suggests that ferroptosis significantly contributes to the pathophysiology of acute tissue injuries, particularly in the lungs [3]. Although many studies have proposed that inhibiting ferroptosis is a novel approach for treating ALI, its broad applicability and specific regulatory mechanisms remain uncertain.

Mild hypothermia has shown definitive efficacy in protecting against neonatal ischemic‒hypoxic encephalopathy and liver‒brain injury following cardiopulmonary resuscitation, as well as in reducing oxidative stress and suppressing inflammation [9]. However, whether mild hypothermia exerts protective effects by inhibiting ferroptosis remains unclear. In this study, we modeled S-ALI in vivo and in vitro, demonstrating that mild hypothermia attenuates iron metamorphosis and oxidative stress through the P53-SLC7A11/GPX4 signaling pathway.

In our study, the SN group exhibited expanded alveolar septa, extensive lung tissue inflammation, increased BALF protein concentrations, increased W/D ratios, decreased pH, PO_2_, PCO_2_, and BE levels, along with significantly increased Lac levels. Compared to the SN group, SH group showed reduced inflammation and improved alveolar structure, with lower total protein concentrations in BALF, higher pH, PO₂, and BE levels, and lower Lac levels. These findings confirm the successful establishment of a sepsis animal model characterized by lung injury and demonstrate that mild hypothermia ameliorates the characteristic symptoms of sepsis.IL-1β and TNF-α are proinflammatory cytokines that initiate systemic inflammatory response syndrome. During the initial stages of sepsis, a cytokine storm ensues as inflammatory substances are released. The SN group demonstrated lower survival rates and higher serum levels of TNF-α, IL-1, and IL-6 compared to the CN group. Conversely, the SH group exhibited decreased TNF-α, IL-1, and IL-6 levels relative to the SN group, leading to improved survival rates. These findings suggest that mild hypothermia alleviates inflammation and enhances survival outcomes in septic rats. Ferroptosis is primarily associated with the disruption of the cellular antioxidant system, meaning that cellular defenses are compromised against free radical attacks and lipid peroxidation [20]. Under normal conditions, oxidation and antioxidant mechanisms are balanced; however, sepsis disrupts this equilibrium, activating both oxidative and antioxidant systems. Fe^2+^ directly promotes ferroptosis and serves as a typical marker of this process [45]. Fe^2+^ accumulation induces lipid peroxidation in cellular membranes, leading to oxidative stress and significant accumulation of ROS and MDA, ultimately resulting in ferroptosis [46]. MDA is a key biomarker reflecting the extent of lipid peroxidation, as it is a byproduct of this process. SOD, a critical antioxidant enzyme, neutralizes superoxide radicals to protect cells from oxidative injury. The combined evaluation of SOD and GSH levels provides a comprehensive indication of an organism's oxidative stress status [47]. Inhibiting ferroptosis mitigates damage to alveolar epithelial cells in ALI[48]. In the S-ALI model, both the SN and SH groups showed increased levels of MDA, Fe^2+^, and ROS, along with decreased SOD and GSH levels compared to the CN group, indicating significant ferroptosis. However, compared to the SN group, the SH group displayed higher SOD and GSH levels, accompanied by lower MDA, Fe^2+^, and ROS levels, suggesting a reduction in ferroptosis. Mild hypothermia treatment decreased ROS production and MDA and Fe^2+^ expression in lung tissues and cells while increasing SOD and GSH expression, indicating significant inhibition of oxidative stress in S-ALI. These findings are consistent with existing literature. Mild hypothermia alleviated inflammatory responses and corrected imbalances in the oxidative and antioxidant systems of S-ALI, thereby mitigating these conditions.

As an upstream transcription factor, p53 regulates the cell cycle, DNA repair, oxidative damage, and ferroptosis. Downregulating SLC7A11 expression reduces cystine uptake [49]. For example, telaprevir downregulates SLC7A11 and GPX4 expression, thereby inducing ferroptosis in osteosarcoma cells. Shi et al [50]. Demonstrated that bavachin triggers ferroptosis in osteosarcoma cells by decreasing SLC7A11 levels and increasing p53 expression [51]. Through the SLC7A11/GPX4 pathway, gentamicin-induced p53 expression may regulate ferroptosis [30]. Iristein upregulates silent information regulator sirtuin 1 expression, which decreases p53 expression and activity, thereby increasing the expression of GPX4 and SLC7A11 and reducing ferroptosis [52]. Research by Chen et al. indicated that independent phospholipase A2 plays a positive role in mitigating p53-triggered ferroptosis and that OS occurs when the formation of ROS surpasses the scavenging ability of the antioxidant system. The lipid-cleansing activity facilitated by iPLA2 is closely linked to the suppression of p53-mediated ferroptosis [53]. Previous studies have established a connection between p53 and ferroptosis, with p53 regulating downstream targets to increase ferroptosis [54]. Our experiments revealed that in rats and cells with S-ALI (SN group), p53 expression was significantly upregulated at both the protein and mRNA levels, while the expression of SLC7A11 and GPX4 was markedly downregulated at both the protein and mRNA levels. Conversely, in the SH group, p53 protein and mRNA levels decreased, and SLC7A11 and GPX4 protein and mRNA levels increased. Mild hypothermic treatment, developed in the early 1980s, has proven to be an effective technique for mitigating central nervous system damage. Zhou et al. reported that controlling the AMPK/NLRP3 pathway decreases ferroptosis-related protein expression and neuroinflammation to alleviate early brain injury following subarachnoid hemorrhage [12]. Studies have demonstrated the advantages of mild hypothermia therapy in reducing acute harm to the liver, kidneys, and lungs resulting from CLP by inhibiting ferroptosis [55]. The mechanisms by which mild hypothermia influences ALI are still under investigation. In our work, mild hypothermia downregulated p53 expression, inhibited SLC7A11 and GPX4, and alleviated ferroptosis. To the best of our knowledge, this study is the first to demonstrate that mild hypothermia mitigates S-ALI by modulating the P53-SLC7A11/GPX4 signaling pathway, leading to a significant reduction in CLP-induced pulmonary edema, oxidative stress, and inflammatory responses.

## Limitations

This study has several limitations. Firstly, we did not employ relevant inhibitors or overexpression agents in our experiments to further validate our findings. Secondly, the mechanisms through which mild hypothermia downregulate p53 remain unexplored. Additionally, our model does not incorporate mechanical ventilation or antibiotic treatment, as their absence may increase mortality rates compared to clinical settings. Furthermore, while there is some basic research and animal experimental data, high-quality clinical studies are still lacking to confirm the therapeutic effects and underlying mechanisms of mild hypothermia in S-AIL. This highlights a gap between experimental outcomes and clinical application.

### Clinical implications

Mild hypothermia has shown promise in several clinical studies for reducing inflammation, alleviating oxidative stress, and improving patient outcomes, particularly in conditions such as severe sepsis, brain injury, and post-cardiac surgery. These findings suggest that a promising therapeutic strategy is mild hypothermia. However, despite supportive clinical evidence, there are challenges and limitations to its application. When considering mild hypothermia for clinical use and trials, we propose the following recommendations: First, mild hypothermia should be viewed as an adjunctive treatment, complementing rather than replacing conventional therapies. Second, the optimal target temperatures and duration of hypothermia should be adjusted according to the severity of conditions such as cerebral edema, hypoxia, or shock. Lastly, due to individual patient differences and the fact that hypothermia treatment is associated with various physiological changes that may lead to complications, it is crucial for the healthcare team to be aware of and anticipate these potential complications. Timely preventive measures, as well as early identification and treatment of complications, can improve overall survival rates.

## Conclusion

This study demonstrates that mild hypothermia mitigates ferroptosis, oxidative stress, and inflammatory responses in S-ALI by modulating the P53-SLC7A11/GPX4 signaling pathway. These findings provide new insights into the pathogenesis of S-ALI and offer scientific support for the development of potential therapeutic interventions targeting ferroptosis and oxidative stress in sepsis-induced organ damage.

## Supplementary Information

Supplementary Material 1.

Supplementary Material 2.

Supplementary Material 3.

## Data Availability

All data generated or analyzed during this study are included in this published article.

## Abbreviations

S-ALI: Sepsis-induced acute lung injury
ALI: Acute lung injury
CLP: Cecal ligation and puncture
LPS: Lipopolysaccharide
GSH: Glutathione
GPX4: Glutathione peroxidase 4
SLC7A11: Solute Carrier Family 7 Member 11
IL: Interleukin
BALF: Bronchoalveolar lavage fluid
MODS: Multiple organ dysfunction syndrome
ROS: Reactive oxygen species
MDA: Malonaldehyde
SOD: Superoxide dismutase
Fe^2+^: Ferrous iron
CCK-8: Cell Counting Kit-8
TNF-α: Tumor necrosis factor-α
