# Supplementary material for: Investigation into the influence of mild hypothermia on regulating ferroptosis through the P53-SLC7A11/GPX4 signaling pathway in sepsis-induced acute lung injury
Source: Intensive Care Med Exp. 2025 Jan 15;13:4. doi: 10.1186/s40635-025-00713-3 (PMC11735705; doi:10.1186/s40635-025-00713-3)
Supplement: Supplementary file 1 — Supplementary Material 1. [file 40635_2025_713_MOESM1_ESM.docx]

**Supplementary Table**

**Lung injury scoring criteria**

| Main features | Score | | | |
| --- | --- | --- | --- | --- |
|  | 0 | 1 | 2 | 3 |
| Edema (number of alveoli involved) | None | 10% | 10-50% | >50% |
| Inflammation (number of inflammatory cells per high magnification view) | None | 10 | 10-50 | >50 |
| Thickening of alveolar septae | None | Mild | Moderate | Severe |
| Alveolar epithelial injury | Absence | Presence |  |  |
