# Supplementary material for: Investigation into the influence of mild hypothermia on regulating ferroptosis through the P53-SLC7A11/GPX4 signaling pathway in sepsis-induced acute lung injury
Source: Intensive Care Med Exp. 2025 Jan 15;13:4. doi: 10.1186/s40635-025-00713-3 (PMC11735705; doi:10.1186/s40635-025-00713-3)
Supplement: Supplementary file 2 — Supplementary Material 2. [file 40635_2025_713_MOESM2_ESM.docx]

**Forward and reverse oligonucleotide sequences of target gene primers.**

| ***Gene*** | ***Primer*** | ***Primer sequences (5′-3′)*** |
| --- | --- | --- |
| P53 | Forward | GGCTCCGACTATACCACTATCC |
|  | Reverse | CAGGCACAAACACGAACCTC |
| GPX4 | Forward | AGGCAGGAGCCAGGAAGTAATC |
|  | Reverse | ACCACGCAGCCGTTCTTATC |
| SLC7A11 | Forward | TATGCTGAATTGGGTACGAGC |
|  | Reverse | TATTACCAGCAGTTCCACCCA |
| IL-1β | Forward | CTCACAGCAGCATCTCGACAAGAG |
|  | Reverse | TCCACGGGCAAGACATAGGTAGC |
| IL-6 | Forward | CTTCCAGCCAGTTGCCTTCTTG |
|  | Reverse | CTTCCAGCCAGTTGCCTTCTTG |
| TNF-α | Forward | CCGATTTGCCATTTCATACCAG |
|  | Reverse | TCACAGAGCAATGACTCCAAAG |
| β-actin | Forward | ACGGTCAGGTCATCACTATCG |
|  | Reverse | GGCATAGAGGTCTTTACGGATG |
