# Supplementary material for: Investigation into the influence of mild hypothermia on regulating ferroptosis through the P53-SLC7A11/GPX4 signaling pathway in sepsis-induced acute lung injury
Source: Intensive Care Med Exp. 2025 Jan 15;13:4. doi: 10.1186/s40635-025-00713-3 (PMC11735705; doi:10.1186/s40635-025-00713-3)
Supplement: Supplementary file 3 — Supplementary Material 3. [file 40635_2025_713_MOESM3_ESM.pdf]

动物实验伦理审查表

The Tab of Animal Experimental Ethical Inspection　　编　号 (No) ：202306672

<table>
<tr><td rowspan="11">申请人填写的相关信息
(Concerned information written by applicant)</td><td colspan="3">申请人(Applicant)：陶柳均</td></tr>
<tr><td colspan="3">申请人学历：硕士研究生
(Education of applicant)：
技术职称：医学生
(Professional title)：</td></tr>
<tr><td colspan="3">实验名称(Study title)：亚低温通过 P53/SLC7A11 信号通路调控铁死亡在脓毒症相关急性肺损伤的作用</td></tr>
<tr><td colspan="3">实验目的(Aim of experiment)：建立动物模型，收集标本。</td></tr>
<tr><td colspan="3">基金来源（Fund sources）：自研课题</td></tr>
<tr><td rowspan="4">拟进动物情况</td><td colspan="2">动物来源(Source of animal)：广西医科大学实验动物中心</td></tr>
<tr><td colspan="2">品种品系(Species or strain)： SD 大鼠　等级(Grade)： spf 级　规格(Specifications)：220-250g</td></tr>
<tr><td>数量(Number)： 24 只(♀ 只；♂ 24 只)</td><td>申请日期(Application date)： 2023 年 6 月 20 日</td></tr>
<tr><td>进驻日期(Entering date)： 2023 年 06 月 21 日</td><td>结束日期(Ending date)： 2024 年 09 月 21 日</td></tr>
<tr><td colspan="3">1.实验要点，实验方法、观测指标
Outline of experiments； experimental methods；observational index：

构建脓毒症大鼠模型，麻醉后解剖大鼠，取肺组织及血液，测炎症、铁死亡指标。

2. 仁慈终点或实验终点：
Human endpoint or experimental terminative indicator
大鼠在解剖采集标本后处死。

3.实验结束后处死动物的方法：
Executing animal method：
腹腔注射巴比妥类药物注射液

4.动物替代、减少动物用量、降低动物痛苦伤害的主要措施等：
Major measure for 3Rs：
科学设计实验方案，熟练掌握实验技术。</td></tr>
<tr><td colspan="3">申请人签名(Signature of applicant)： 陶柳均　　联系电话(Telephone)：15506779511</td></tr>
<tr><td>审查结果
(是否同意</td><td colspan="3">课题负责人意见
(Study director)：　同意 (Agree) ☑　不同意 (Disagree) ☐　签　名：汤展宏 (Signature)</td></tr>
</table>

<table>
<tr><td rowspan="2">申请人的实验方案)
(Results of inspection)</td><td>实验动物设施意见(Opinion from laboratory animal facility)：

同意(Agree) ☑ 不同意(Disagree) ☐

签　　名
(Signature)</td></tr>
<tr><td>实验动物福利与伦理委员会意见(The Animal Care & Welfare Committee)：

同意(Agree) ☑ 不同意(Disagree) ☐

签　　章
(Stamp)</td></tr>
<tr><td colspan="2">备注(Supplement)：
初审 ☑ First trial　　　　第　　次审查 reexamine No.</td></tr>
</table>

填表须知：动物伦理审查遵循中华人民共和国科技部发布的《关于善待实验动物的指导性意见》及中华人民共和国国家标准 GB/T35892-2018《实验动物 福利伦理审查指南》，如有不明确之处请参看这两份文件或咨询广西医科大学实验动物福利与伦理委员会。

Notes : Animal ethics review follows the *Guiding Opinions on the Treatment of Laboratory Animals* issued by the Ministry of Science and Technology of the People's Republic of China and the *Laboratory Animal-Guideline for Ethical Review of Animal Welfare* issued by the National Standard GB/T35892-2018 of the People's Republic of China. If there are any uncertainties, please refer to these two documents or consult The Animal Care & Welfare Committee of Guangxi Medical University.
